# Isolation and functional characterization of a cotton ubiquitination-related promoter and 5'UTR that drives high levels of expression in root and flower tissues

**DOI:** 10.1186/1472-6750-11-115

**Published:** 2011-11-24

**Authors:** Antonio AB Viana, Rodrigo R Fragoso, Luciane M Guimarães, Naiara Pontes, Osmundo B Oliveira-Neto, Sinara Artico, Sarah M Nardeli, Marcio Alves-Ferreira, João AN Batista, Maria CM Silva, Maria F Grossi-de-Sa

**Affiliations:** 1 Laboratório de Interação Molecular Planta-Praga, Embrapa Recursos Genéticos e Biotecnologia, PqEB final W5 Norte, Brasília/DF, 70770-900, Brasil; 2 Universidade Católica de Brasília, QS 07 Lote 01 EPCT, Taguatinga/DF, 71966-700, Brasil; 3 Embrapa Cerrados, Rodovia Brasília/Fortaleza BR 020, Km18, Planaltina/DF, 73310-970, Brasil; 4 Depto. Biologia Celular, Universidade de Brasília, IB, Campus Universitário Darcy Ribeiro, Brasília/DF, 70910-900, Brasil; 5 Depto. Genética, Universidade Federal do Rio de Janeiro, Centro de Ciências da Saúde (CCS), Bloco A, 2° andar, Sala 85, Ilha do Fundão, Rio de Janeiro/RJ, 21941-570, Brasil; 6 Depto. Botânica, Universidade Federal de Minas Gerais, Instituto de Ciências Biológicas, Av. Antônio Carlos 6627, Pampulha, Belo Horizonte/MG, 31270-901, Brasil

## Abstract

**Background:**

Cotton (*Gossypium *spp.) is an important crop worldwide that provides raw material to 40% of the textile fiber industry. Important traits have been studied aiming the development of genetically modified crops including resistance to insect and diseases, and tolerance to drought, cold and herbicide. Therefore, the characterization of promoters and regulatory regions is also important to achieve high gene expression and/or a specific expression pattern. Commonly, genes involved in ubiquitination pathways are highly and differentially expressed. In this study, we analyzed the expression of a cotton ubiquitin-conjugating enzyme (E2) family member with no previous characterization.

**Results:**

Nucleotide analysis revealed high identity with cotton *E2 *homologues. Multiple alignment showed a premature stop codon, which prevents the encoding of the conserved cysteine residue at the *E2 *active site, and an intron that is spliced in *E2 *homologues, but not in *GhGDRP85*. The *GhGDRP85 *gene is highly expressed in different organs of cotton plants, and has high transcript levels in roots. Its promoter (uceApro2) and the 5'UTR compose a regulatory region named uceA1.7, and were isolated from cotton and studied in *Arabidopsis thaliana*. uceA1.7 shows strong expression levels, equaling or surpassing the expression levels of CaMV35S. The uceA1.7 regulatory sequence drives GUS expression 7-fold higher in flowers, 2-fold in roots and at similar levels in leaves and stems. GUS expression levels are decreased 7- to 15-fold when its 5'UTR is absent in uceApro2.

**Conclusions:**

uceA1.7 is a strong constitutive regulatory sequence composed of a promoter (uceApro2) and its 5'UTR that will be useful in genetic transformation of dicots, having high potential to drive high levels of transgene expression in crops, particularly for traits desirable in flower and root tissues.

## Background

Cotton plants (*Gossypium *spp.) produce the most widely used textile fiber in the world. Moreover, the cotton seed is used to feed livestock and its oil is important in biodiesel production and human consumption. In 2010, more than half world cotton area was planted with genetically modified (GM) cotton, which occupied 21.0 million hectares (14% global biotech area) [1]. Up to date, around 35 cotton events have been approved, in which insect-resistant trait is one of most used and spread, behind only of herbicide tolerance or both traits combined [1].

Despite significant efforts towards the isolation and characterization of plant genes, only a reduced number of plant promoters have been isolated and functionally characterized [2-4]. The most widely used general-purpose promoter in GM plants is the Cauliflower Mosaic Virus (CaMV) 35S promoter [5], used in more than 80% of GM plants [6] because of its constitutively high levels of transgene expression [7]. However, the stability and expression patterns of foreign genes driven by the CaMV35S promoter, including genes expressed in cotton [8,9], have been widely debated [9-11]. Moreover, the combination of different traits like drought and cold tolerance, bacteria, fungi and nematode resistance, requires different promoter activities not only to avoid transgene silencing, but also to target the expression to different tissues or developmental stages. Up to date, around 15 different promoters have been used and evaluated in GM cotton varieties, usually chimeric products from viral and/or *Agrobacterium *promoters, with 5 being derived from maize or *Arabidopsis *promoters [12]. Therefore, the discovery and characterization of additional plant promoters is essential to a better understanding of transgene expression in GM plant development with predictable high level, temporal and tissue-specific expression patterns [13].

The ubiquitination-related genes have been reported to display high levels of expression across several plant tissues [14], provided that ubiquitination is a major posttranslational protein modification, which is based on the addition of one or multiple ubiquitin molecules to the lysine residues of substrate proteins [15]. Ubiquitination plays important roles in the regulation of lipidation, protein activity, protein-protein interactions, subcellular protein localization [16], transcriptional regulation (through histone ubiquitination), translational regulation, DNA repair, endocytosis and protein turnover and trafficking [17].

Ubiquitination-related gene promoters isolated from *Arabidopsis *[18], sunflower [19], tobacco [20], maize [21] and several other crops can drive the expression of reporter genes in transformed cells or plants. Previous studies have reported that the expression pattern of the ubiquitin-conjugating enzymes (E2s) is differentially regulated across tissues and developmental stages [22-24].

Therefore, the main goal of this study is the evaluation of the expression pattern of a non-characterized cotton ubiquitin-conjugating enzyme (E2)-related gene in order to isolate a novel promoter with different properties. In this study, we report the identification of the gland development related protein 85 [GenBank:EU373075] *GhGDRP85 *gene, a member of the E2 family that presents a high level of transcript accumulation in several cotton tissues. The *GhGDRP85 *regulatory region, hereby named uceA1.7 [GenBank:JN887312] and promoter named uceApro2 [GenBank:JN887311] was isolated and its GUS expression pattern characterized in transgenic *Arabidopsis*. The data generated in this study indicate that uceA1.7 has potential to be a reliable alternative to the CaMV35S promoter and its variations to be applied in GM crop generation programs.

## Results

### Root preferential expression of an ubiquitin-conjugating enzyme related gene in cotton plants

Aiming the identification and the isolation of a novel cotton promoter, a search was performed in the GenBank for the ubiquitin-conjugating enzyme (E2) family members. The search resulted in seven E2 sequences, retrieved from four different cotton species. A multiple sequence alignment analysis revealed that six of the identified sequences are closely related with high scores (>1,000) and low e-values (<1.00E-24). However, one sequence deposited as '*Gossypium hirsutum *gland development related protein 85-like mRNA' (GhGDRP85, [GenBank:EU373075.1]) showed a slightly lower score and a higher e-value than all the analyzed E2 sequences. No reports were found in regards to GhGDRP85 protein. Due to its relatively high identity with the cotton E2s (e-values ranging from 1.00E-24 to 1.00E-55), we selected it as a good candidate and carried out further analysis.

**Figure 1:**
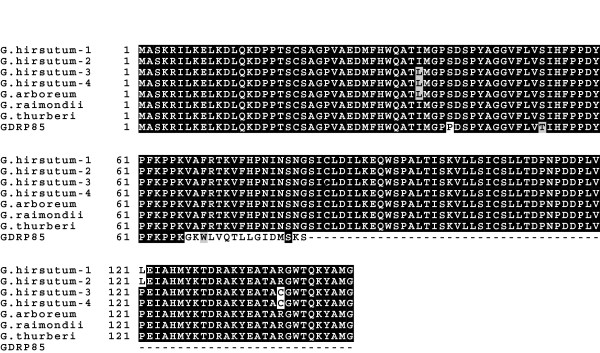
**Multiple protein sequence alignment of the E2 family members in cotton**. *G. hirsutum*-1 to 4 correspond to the protein sequences [GenBank:AAL99220.1, GenBank:AAL99222.1, GenBank:AAL99221.1 and GenBank:AAL99219.1] [45]. *G. raimondii*, *G. thurberi*, *G. arboreum *and GhGDRP85 [GenBank:AAL99225.1, GenBank:AAL99224.1, GenBank:AAL99223.1 and GenBank:EU373075.1], respectively. The cysteine residue required for ubiquitin binding is indicated by an asterisk. The alignment was performed using ClustalW [55] and identity/similarity shading was performed with BoxShade 3.21.

Multiple nucleotide sequence alignment showed a 247-bp insertion in *GhGDRP85 *gene (Additional file 1 Figure S1). The nucleotides flanking the insertion are similar to exon-exon junctions in all six other analyzed E2 sequences, which strongly suggests that an intron in *GhGDRP85 *is not removed during RNA splicing. Furthermore, the putative not processed intron includes a stop codon rendering a truncated E2 homologue, missing the active cysteine residue site (Figure 1), which may block ubiquitin transfer. This observation prompted us to investigate the expression pattern of the *GhGDRP85 *gene.

Specific primers for qPCR were designed, in which the reverse primer annealed to the putative not processed intron (Additional file 1 Figure S1), allowing us to analyze the possibility of its retention and to assay the expression pattern of *GhGDRP85 *gene without interference from the other homologue mRNAs that share high sequence identity.

**Figure 2:**
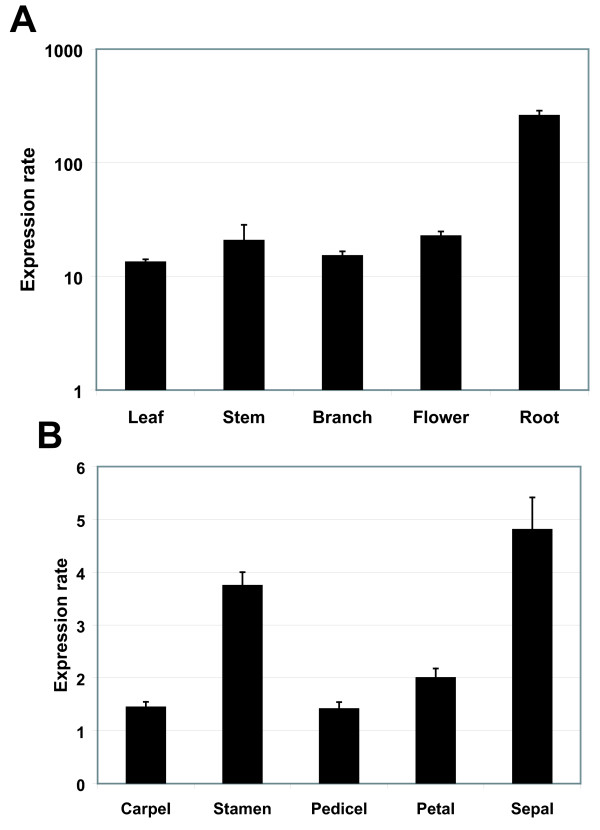
**Expression profiles of the *GhGDRP85 *in cotton plants**. The transcript levels of different cotton organs and tissues were determined by qPCR. (**A**) Log scale of the transcript levels of the *GhGDRP85 *gene normalized with the reference genes *GhPP2A1 *and *GhUBQ14 *in several plant organs. (**B**) Transcript levels of the *GhGDRP85 *gene in floral tissue relative to the reference genes *GhACT4 *and *GhFBX6*.

The qPCR analysis (Figure 2A) employed *GhUBQ14 *and *GhPP2A1 *as reference genes (Additional file 2 Table S1) because of their high stability [25]. *GhGDRP85 *transcripts are at least 10 times more abundant than reference genes in all analyzed tissues. In roots, *GhGDRP85 *transcripts accumulated approximately 200-fold more than the reference genes.

Due to the fact that cotton flowers are targets of severe cotton pests, such as the cotton boll weevil (*Anthonomus grandis*), it was necessary to gain information in regards to the expression patterns in floral organs. The four floral verticils (carpel, stamen, petal and sepal), as well as the pedicel of 6-mm flower buds, were harvested and dissected. In this case, the data were normalized to the *GhACT4 *and *GhFBX6 *reference genes due to its higher stability in flower tissues [25]. The analysis revealed similar *GhGDRP85 *transcript levels in all flower verticils, indicating its regulatory region as a potential alternative to the CaMV35S to direct strong gene expression to floral tissues (Figure 2B).

### Isolation of the uceApro2 promoter and its 5'UTR from cotton plants

Because of the *GhGDRP85 *interesting expression pattern, we proceeded with the isolation of its regulatory region as a potential candidate for biotechnological use. The regulatory sequence upstream to *GhGDRP85 *was amplified by TAIL-PCR [26], using alternating rounds of high and low stringency to amplify specific products preferentially over non-specific products. An 1,049-kb fragment named uceA1.7 [GenBank:JN887312] was cloned (Additional file 3, Figure S2) and sequenced. Alignment of this cloned sequence showed an overlap of the 3' end of the isolated sequence with the 5' end of the *GhGDRP85 *mRNA. The 5' end of the cloned sequence contained no open reading frames and no significant identity to any previously described promoters.

**Figure 3:**
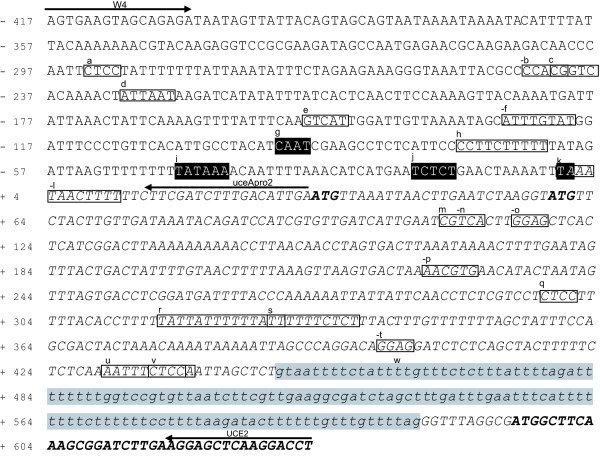
**Nucleotide sequence of the cotton uceA1.7 regulatory region and uceApro2 promoter**. The nucleotides are numbered with respect to the predicted transcription start site (TSS), taken as +1. Italicized characters are used for the transcribed sequence. Cis-acting sequence motifs identified by PlantCARE software are boxed (listed in Table 1) and identified by letters (**a **to **w)**. Minus signs denote reverse and complement sequence motifs. The predicted core promoter motifs, CAAT Box (**g**), TATA Box (**i**), Y Patch (**j**) and YR Rule (**k**), are black shaded and in white letters. An intron in 5'UTR is gray shaded with lowercase letters. Translated *GhGDRP85 *sequence is in bold. The annealing regions of W4, UCE2 and uceApro2 primers are shown with arrows. The uceApro2 promoter corresponds to the nucleotide region -417 to +31.

The sequence of the isolated regulatory region was analyzed to predict the core promoter and cis-elements using the PlantCARE software (Figure 3). PlantCARE predicted four CAAT-boxes and 19 TATA-boxes. The most probable combination of CAAT-box and TATA-box was selected according to the common promoter pattern, also considering predicted transcription start site (TSS), following the Y Patch and YR Rules [27]. Using this strategy, the region of the core promoter was therefore predicted (shaded in black in Figure 3) as one functional group. Based on these predictions, the core promoter plus its upstream 325-bp region was isolated and named uceApro2 [GenBank:JN887311]. The rationale behind the isolation of these two sequences was the assessment of the 5'UTR influence in the expression pattern of the uceApro2 promoter, provided that 5'UTRs have been reported to enhance the expression levels of reporter genes in previous studies [28,29]. In addition, uceA1.7 contains several putative functional cis-regulatory elements, boxed in Figure 3 and briefly described in Table 1.

**Table 1:** Sequence motifs of cis-acting elements found in uceA1

Motif sequence (letter in Figure 3)	Motif name	Motif site (from/to)	Strand	Expression
(**a**) CTCC	Unnamed 4	-293/-290	+	Unknown
(**b**) CGTGG	Unnamed1/3	-245/-241	-	Unknown
(**c**) CGGTCA	MBS	-242/-237	+	MYB Binding Site; Weak homology to the consensus TAACTG/CAGTTA involved in Bz2 gene activation and R and C1 factors
(**d**) ATTAAT	Box 4	-229/-224	+	Part of the pal conserved DNA module array (pal-CMA1) involved in light responsiveness
(**e**) GTCAT	Skn-1 motif	-149/-145	+	Required for high levels of endosperm expression in cooperative interaction with others motifs (AACA, GCN4, ACGT)
(**f**) ATACAAAT	ATGCAAAT-motif	-127/-120	-	One base mismatch; Associated to the TGAGTCA motif (GCN4) separated by two bases in tandem
(**g**) CAAT	CAAT-box (4)	-92/-89	+	Core promoter; common element in promoter and enhancer regions; about 50 bp from TATA-box
(**h**) CCATCTTTTT	TCA-element	-72/-63	+	Involved in salicylic acid responsiveness; one TCA-element does not confer salicylic acid responsiveness, more than one might do it
(**i**) TTATAAAA	TATA-box (19)	-44/-37	+	Core promoter; element found from -50 to -20 of the TSS
(**j**) TCTCT	Y Patch	-17/-13	+	Core promoter; found between -100 to -1 of TSS
(**k**) Pyrimidine/Purine	YR Rule	-1/+1	+	Core promoter; Transcription Start Site (TSS)
(**l**) AAAAGTTAGTTA	MBSII	-1/+11	-	MYB binding site involved in flavonoid biosynthetic genes regulation
(**m**) CGTCA	CGTCA-motif	+107/+111	+	Involved in the MeJA-responsiveness; associated with its complementary sequence TGACG 15 bp downstream (palindrome)
(**n**) TGACG	TGACG-motif	+107/+111	-	cis-acting regulatory element involved in the MeJA-responsiveness
(**o**) CTCC	Unnamed 4	+115/+118	-	Unknown
(**p**) CACGTT	G-Box	+225/+230	-	cis-acting regulatory element involved in light responsiveness light responsive element site associated to Box II and P-box to confer elicitor-mediated gene activation in a number of phenylpropanoid genes
(**q**) CTCC	Unnamed 4	+298/+301	+	Unknown
(**r**) AATTATTTTTTATT	AT1-motif	+316/+329	+	Part of light responsive module
(**s**) TTTCTTCTCT	5'UTR Py-rich stretch	+328/+339	+	Region in the 5'UTR conferring high transcription levels without the need for other upstream cis elements except for a TATA-box
(**t**) CTCC	Unnamed 4	+400/+403	-	Unknown
(**u**) ATTTTCTCCA	TC-rich repeats	+431/+439	+	cis-acting element involved in defense and stress responsiveness
(**v**) CTCC	Unnamed 4	+435/+438	+	Unknown
(**w**) AGAAACAA	AE-box	+462/+469	-	part of a system composed of 3 to 4 Gap-boxes and 2 AE-boxes, conferring light responsiveness

Sequence motifs of the cis-acting promoter found in uceA1.7 obtained using PlantCARE software [1].

The sequence alignment between uceA1.7 and the *GhGDRP85 *mRNA reveals the existence of an intron in the 5'UTR (shaded in gray in Figure 3). This intron is flanked by nucleotides that match with junction motifs, and the intronic sequence is spliced 10 bp upstream of the translation initiation codon. The presence of an intron in the *GhGDRP85 *5'UTR provides an indication that it may contain important cis-elements involved on intron-mediated enhancement (IME) of gene expression [30,31].

### Functional characterization of uceA1.7

**Figure 4:**
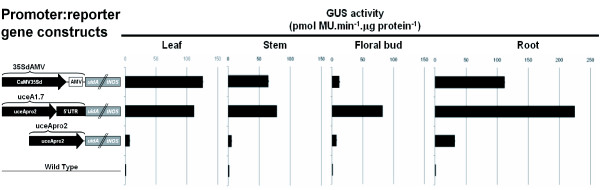
**Schematic representation of the promoter:reporter gene constructs and GUS activity in different plant tissues**. The uceA1.7 and uceApro2 constructs were sub-cloned and compared to the commonly used CaMV35Sd plant promoter. GUS specific activity was measured by a fluorometric assay in *A. thaliana *leaves, stems, floral buds and roots. CaMV35Sd: modified viral constitutive promoter; AMV: viral 5'UTR enhancer; *uidA//tNOS*: GUS gene with the NOS terminator; uceA1.7: *GhGDRP85 *regulatory sequence containing uceApro2, and the 5'UTR of *GhGDRP8*; uceApro2: *GhGDRP85 *gene promoter. The figure elements are not to scale.

To analyze the tissue expression pattern directed by the uceA1.7 regulatory sequence in a heterologous system, GM *Arabidopsis *plants harboring three different promoter:reporter gene constructs were generated. The duplicated CaMV35S with the alfalfa mosaic virus enhancer (35SdAMV) [32], uceA1.7 and uceApro2 were individually inserted into pCAMBIA1391 upstream of the *uidA *gene coding region followed by the tNOS terminator (Figure 4). A quantitative assessment of β-glucuronidase (GUS) activity was measured using a fluorometric assay with protein extracts from leaf, stem, floral bud and root tissues. All three sequences drove GUS expression in all four tissues examined (Figure 4).

**Figure 5:**
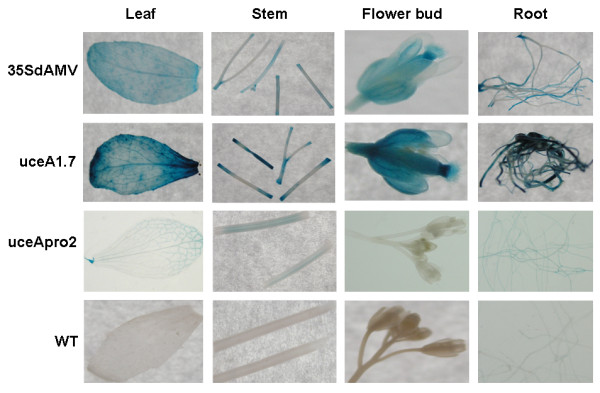
**Histochemical assays showing GUS expression under the control of different regulatory regions in several *Arabidopsis thaliana *tissues**. Samples of leaves, stems, flower buds and roots of *A. thaliana *expressing the *uidA *gene under control of the 35SdAMV, uceA1.7 and uceApro2 nucleotide sequences.

The levels of GUS activity directed by uceA1.7 and 35SdAMV were quite similar in leaf (a ratio of 0.9) and stem (a ratio of 1.2) tissues. However, uceA1.7 drove a 2-fold higher level of GUS expression in roots and about 7-fold higher level in floral buds compared to the widely used 35SdAMV (Figure 4 and 5). Moreover, uceA1.7 drove around 15, 13, 11 and 7-fold higher expression levels in leaves, stems, floral buds and roots, respectively, compared to uceApro2. The uceApro2 promoter directed basal GUS expression mainly in vascular tissues, such as the vascular cambia and leaf veins (Figure 5), whereas uceA1.7 showed strong and well-distributed expression in all tissues and organs.

## Discussion

Ubiquitin-conjugating enzymes (E2) are known to show both spatial and temporal regulated expression, because their role in ubiquitination process, which can be associated to different molecular, cellular and physiological processes. With the goal of identifying a promoter and/or a regulatory region in cotton with new properties, we first analyzed *Gossypium *spp E2 family in GenBank. Among the seven members found, one of them (GhGDRP85) seems to be a truncated version of the E2 homologues due to a premature stop codon. The stop codon is present in an uncommon region that seems to be an unspliced intron. Indeed, the *GhGDRP85 *mRNA possesses an internal 274-bp sequence that is spliced as an intron in an alternative *Gossypium E2 *transcript. This data was confirmed by qPCR using a reverse primer annealing in this region. The premature termination of GhGDRP85 protein prevents the conserved cysteine residue (Cys85) of the active site responsible for ubiquitin transfer to be encoded [33]. The truncated E2 protein may act as a competitive inhibitor of the functional E2, and thereby down-regulate the ubiquitination pathway. Therefore, it seems that GhGDRP85 protein has evolved from E2 family, but as a result of alternative splicing that ultimately encodes a truncated E2-related protein. We observed intron junctions that could alternatively spliced over this 274-bp intron from the *GhGDRP85 *primary transcript under specific conditions, developmental stages and/or in certain tissue types. The alternative mRNA encodes the Cys85 along an E2 protein that shares very high sequence identity (from 2E-98 to 3E-109, 97-100%) with E2 family members, including UBC2, 8, 9, 10, 11, 28 and 30.

Thus, the *GhGDRP85 *transcript abundance in cotton tissues was evaluated to verify its regulatory sequence activity and its biotechnology usefulness. The *GhGDRP85 *transcript abundance was approximately 10-fold greater than the reference genes *GhPP2A1 *and *GhUBQ14 *in four analyzed tissues (leaf, stem, branch and flower). These results showed that the *GhGDRP85 *transcript is highly abundant in most cotton tissues, especially in roots, which the mRNA levels are 200-fold higher than the reference genes (Figure 2).

To obtain further detail about the expression activity of the *GhGDRP85 *regulatory region, we isolated the corresponding upstream promoter (uceApro2) within the *GhGDRP85 *regulatory region (which included uceApro2 and a 5'UTR, named uceA1.7). The activity and the spatial gene expression driven by the constructs uceA1.7, uceApro2 and 35SdAMV nucleotide sequences fused to the GUS reporter were evaluated in the respective transgenic *Arabidopsis *plants.

Transgenic plants harboring uceApro2::GUS present basal levels of GUS activity in leaf, stem, flower bud and root, although GUS activity in root was approximately 4-fold higher when compared to other tissues (Figure 4). The PlantCARE software did not identify any cis-acting element that would explain such high levels of expression in roots (Table 1). Therefore, further analyses are still needed to locate root-specific cis-acting elements.

The remarkable difference in expression levels (Figures 4 and 5) driven by the uceA1.7 and uceApro2 constructs can be directly associated to the presence of the *GhGDRP85 *5'UTR. The presence of this region triggers an important quantitative effect, increasing from 11 to 15-fold expression in leaf, stem and flower bud tissues. In root the presence the 5'UTR result in an increase of only 7-folds, however it is also possible that the detection sensitivity of the GUS assay has reached its saturation point in the root tissues. On the other hand, some putative light-responsive cis-acting elements observed in 5'UTR could be responsible for the higher GUS expression in light-exposed tissues (motifs (p) and (r) in Figure 3 and Table 1).

In addition to the numerous regulatory elements present along the 5'UTR to regulate the transcription process, this sequence may also possess other elements that increase favorable effects on mRNA stability, processing, nucleus-cytoplasm translocation and translational apparatus assembly [13,34,35], such as an intron just before the first exon that could characterize intron-mediated enhancement (IME) [30,31]. Taken together, these findings suggest that the presence of the 5'UTR downstream of the uceApro2 promoter likely accounts for the observed increase in expression levels driven by uceA1.7. This phenomenon has already been described in previous reports of gene expression analyses in pea and conifers [28,29].

The uceA1.7 sequence drives GUS expression to leaves and stems in comparable levels to the strong constitutive enhanced plant promoter CaMV35S. However, uceA1.7 drives higher GUS expression in roots (2-fold) and flowers (7-fold). 35SdAMV:GUS plants present a 10-fold decrease of GUS expression in flower bud, relative to leaf. It is noteworthy that GUS staining in leaf (Figure 5) driven by uceA1.7 seems to be higher than that driven by CaMV35S. However this observation does not match to the quantitative fluorometric data presented on Figure 4. This kind of variance is commonly observed with GUS staining assays. Furthermore, the CaMV35S promoter, which has been widely used for plant expression systems, induces low and variable expression in floral organs [9], rendering the production of plants expressing foreign genes on floral organs unpredictable. Due to the reported reduced gene expression driven by CaMV35S promoter in different plants upon nematode infection [36], we intend to perform the analyses of uceA1.7 driven expression at nematode feeding sites.

The *GhGDRP85 *expression patterns in cotton plants are comparable to the GUS expression assays in transgenic *A. thaliana *plants. Despite of the similarity of expression levels measured in leaf, stem and flower tissues, the levels of *GhGDRP85 *transcript abundance in roots were much higher than the levels of GUS measured in the fluorometric assays. If there is no GUS detection limit threshold, we suggest that this difference might be due to the absence of cotton-specific trans-acting factors in *Arabidopsis*. Alternatively, the cloned uceA1.7 sequence might not contain cis-acting enhancer elements that are located far up- or downstream and present in the native *GhGDRP85 *gene regulatory region.

## Conclusions

In conclusion, the uceA1.7 regulatory sequence isolated in this study drives high and constitutive expression in *Arabidopsi*s plants and displays great potential to be applied as biotechnological tool for the generation of GM crops, especially cotton. This sequence is particularly promising for those traits requiring high expression levels in root and flower tissues.

## Methods

### Cotton plant material for qPCR

Three-month-old, greenhouse-grown *Gossypium hirsutum *plants (BRS Cedro) were used in this study. Flower buds (2-10 mm), leaves, stems, branches, roots and floral organs (sepal, petal, stamen, carpel and pedicel; from 6 mm flower buds) were harvested from at least five different plants and pooled. The entire procedure was repeated to obtain a second pool. All samples were immediately frozen in liquid nitrogen and stored at -80°C until RNA extraction.

### Real-time quantitative polymerase chain reaction (qPCR)

Total RNA was extracted from 100 mg of ground plant tissue using the Invisorb Spin Plant RNA Mini kit (Invitek), according to the manufacturer's instructions. RNA concentration and purity were determined using a NanoDropTM Spectrophotometer ND-1000 (Thermo Scientific). The integrity of the RNA was also assessed by 1% agarose gel electrophoresis. The presence of contaminating DNA in the RNA samples was tested by PCR and gel electrophoresis analysis. No genomic DNA was identified in any of the samples tested (data not shown). The presence of spurious amplification products caused by genomic DNA was also continuously monitored by the verification of the qPCR dissociation profile. The cDNA synthesis was performed with 1 μg of total RNA using the Superscript III kit (Invitrogen), according to the manufacturer's protocol.

Primers for qPCR were designed with Primer 3 software [37] using the criteria for generating amplified products with sizes ranging from 80 to 180 bp, with a T_m _of approximately 60°C, resulting in RT_uceA1_7-For sense primer (5' ATATGCCGGTGGAGTGTTTC 3') and RT_uceA1_7-Rever antisense primer (5' TCATGTCAATGCCAAGCAAT 3'). Primers for reference genes were the same used in our previous study [25]. Primer set efficiencies were estimated for each experimental set with the *Miner *software [38], and the values were used for all subsequent analyses. PCRs were carried out in an optical 96-well plate with a Chromo4 Real time PCR Detector sequence detection system (BioRad) using SYBR^®^Green to monitor dsDNA synthesis. The reaction mixture contained 10 μl of diluted cDNA (1:50), 0.2 μM of each primer, 50 μM of each dNTP, 1X PCR Buffer (Invitrogen), 3 mM MgCl_2_, 1 μl SYBR^®^Green I (1:10,000 dilution in water; Molecular Probes) and 0.25 U of Platinum Taq DNA polymerase (Invitrogen) in a total volume of 20 μl. Reactions were incubated for five min at 94°C, followed by 40 amplification cycles of 15 s at 94°C, 10 s at 60°C and 15 s at 72°C. PCR efficiencies and optimal cycle threshold (Ct) values were estimated using the Realtime PCR *Miner *[38]. Ct values were converted in qBase software v1.3.5 [39] into non-normalized relative quantities, corrected by PCR efficiency, using the formula Q = E^ΔCt ^where E is the efficiency of the gene amplification and ΔCt is the sample with the lowest expression in the data set minus the Ct value of the sample in question. *GhUBQ14 *and *GhPP2A1 *were used as reference genes for the normalization of qPCR data for plant organs (leaf, stem, branch, flower bud and root) and *GhACT4 *and *GhFBX6 *as reference genes for floral organs (carpel, stamen, pedicel, petal and sepal). The identification of the ideal reference genes was previously performed [25].

### Isolation of the uceA1.7 sequence from cotton plants

The regulatory region of the *GhGDRP85 *gene was isolated from plant genomic DNA using the TAIL-PCR technique [26]. Two PCR rounds (primary and secondary) were performed sequentially using two antisense primers based on the sequence of the *GhGDRP85 *gene 5' terminus sequence and a random sense primer against the unknown sequence of the promoter region. Briefly, DNA extraction was performed using the DNeasy plant mini kit (QIAGEN), and the promoter was amplified as follows: *(i) Primary reaction*: 200 ng of genomic DNA (cotton cv. IAC98/708) was added to a 20-μl PCR mix containing 20 mM Tris-HCl, 50 mM KCl, pH 8.4, (Invitrogen PCR buffer), 2 mM MgCl_2_, 200 μM dNTPs, 400 nM primer UCE1 (5' GCTTGCCAATGGAACAT 3'), 3 μM primer W4 (5' AGWGNAGWANCANAGA 3') and 1 U Taq DNA polymerase (Invitrogen). The reaction had an initial denaturation step of 2 min at 94°C, 5 cycles of 1 min at 94°C, 1 min at 55°C and 2.5 min at 72°C, followed by a denaturation step of 1 min at 94°C, a temperature ramp ranging from 25 to 72°C during 3 min and an extension of 2.5 min at 72°C. The reaction progressed through 15 cycles consisting of two incubations of 30 s at 94°C, 1 min at 60°C, 2.5 min at 72°C (two high stringency cycles) and one incubation of 30 s at 94°C, 1 min at 44°C, 2.5 min at 72°C (one low stringency cycle), and a final extension at 72°C for 5 min; *(ii) Secondary reaction*: 1 μl of a 1:50 dilution of the primary reaction product was added to 19 μl of a similar PCR mix, with the exception that 400 nM primer UCE2 (5' AGRTCCTTIAGCTCCTT 3'), 2 μM primer W4 and 0.6 U Taq DNA polymerase (Invitrogen) were used. Control reactions were performed using the W4 primer and the UCE2 primer only. The reactions were run for 12 cycles of two incubations of 30 s at 94°C, 1 min at 55°C and 2.5 min at 72°C (two high stringency cycles) followed by one incubation of 94°C for 30 s, 44°C for 1 min, 72°C for 2.5 min (one low stringency cycle), and a final extension at 72°C for 5 min.

The PCR products were separated by TBE agarose gel electrophoresis, and the amplified fragment was purified from the gel using the GeneClean II kit (Bio 101), according to the manufacturer's instructions. The fragment was then sub-cloned into the pGEM-T vector (Promega), and the recombinant vector was used to transform *Escherichia coli *cells by electroporation. Colonies were selected by beta-complementation, and small-scale plasmid DNA were extracted as described [40] for further sequencing using T7 and SP6 primers.

### Sequence analysis of the uceA1.7

The complete isolated sequence of uceA1.7 was analyzed with the PlantCARE [2], PLACE [41,42] and PlantPAN [43] software packages. The Y Patch and YR Rule were visually selected from several possibilities [27]. This allowed us to suggest the 5'UTR and isolate the uceApro2 promoter.

### Binary vectors cloning

The uceA1.7, uceApro2 and 35SdAMV sequences were sub-cloned from pGEM-T into the plant transforming binary vector pCAMBIA1391 upstream of the *uidA *coding sequence, followed by the termination sequence from the nopaline synthase gene of *Agrobacterium *(tNOS). Both vectors were double-digested with *Nco*I and *Spe*I. The resulting fragments were purified with the GeneClean II kit (Bio 101), cloned using the T4 DNA ligase system and used to transform *E. coli *cells by electroporation. Clones were selected and analyzed by colony PCR using the W4 and UCE2 primers. The pCAMBIA1391/uceA1.7 construct includes the uceApro2 promoter, the 5'UTR and 38-bp region of the first exon of the *GhGDRP85 *gene translationally fused with the *uidA *reporter gene.

The uceApro2 promoter was directly cloned upstream to the *uidA *reporter gene as described above. After PCR amplification with a specific antisense primer uceApro2-R (5' CAATGTCAAAGATCGAAG 3') located at +14/+31, 68 bp downstream from the predicted TATA-box, the product was cloned into pGEM-T and sub-cloned into pCAMBIA1391, as described previously.

For comparison of expression levels and patterns, the 35SdAMV promoter was used. This construct, which consists of the duplicated CaMV35S [44] with the AMV (Alfalfa Mosaic Virus) 5'UTR enhancer [39,45-47], was sub-cloned into pCAMBIA1391, using the same methods described previously.

### Plant transformation

The three vector constructs (1 μg each) were used to transform *Agrobacterium tumefaciens *LBA4404 via heat-shock (30 min at 0°C, 5 min at 37°C and 2 h at 28°C in YEB medium) [48]. The transformed *Agrobacterium *cells were then used to transform *Arabidopsis thaliana *(Columbia) floral buds via the floral dip infiltration method [49,50]. The seeds (T1) of the transformed plants were selected in MS media [51] supplemented with 20 μg/ml hygromycin. Plantlets were transferred to a greenhouse for further analyses.

### Fluorometric and histochemical assays for GUS activity

Functional characterization of uceA1.7 and uceApro2 was performed using *Arabidopsis *plants grown in greenhouse conditions for six weeks. Sections of leaves, stems, flower buds and roots were collected from at least three plants for each construct, pooled and ground in protein extraction buffer (100 mM phosphate buffer pH 7.0, 10 mM EDTA, 0.1% Triton X-100, 0.1% Sarcosyl, 1 mM DTT, 25 μg/ml PMSF and 10 μg/ml leupeptin) then centrifuged at 12,000 × g for 5 min at 4°C. Protein extracts were quantified [52], and 50 μl of each extract was added to 100 μl of substrate solution containing 2 mM MUG (4-methyl-umbelliferyl-glucuronide) in protein extraction buffer followed by incubation at 37°C. At different time points (0, 15 and 30 min), an aliquot of 40 μl of the reaction was quenched with 200 mM Na_2_CO_3_. After the third time point, the fluorescence emission of all time points was measured using three replicates.

*Arabidopsis *tissue samples (leaf, stem, floral bud and root) for each construct (35SdAMV, uceA1.7 and uceApro2) and WT (wild type plants) were collected and analyzed for GUS expression. The samples were incubated in a 2 mM X-gluc solution [53] for 16 hours at 37°C as described by Howard and colleagues [54] and analyzed using optical microscopy.

## Authors' contributions

AABV participated in the study design, cloning, designed and performed *Arabidopsis *transformation, histochemical assays, fluorimetric assays, manuscript writing, data interpretation and revision. RRF participated in experimental design, sequence analyses, histochemical assays, qPCR, manuscript writing, data interpretation, discussion and revision. LMG participated in cloning, *Arabidopsis *transformation, histochemical assays and fluorimetric assays. NP worked on *Arabidopsis *transformation, histochemical assays and fluorimetric assays. OBON participated in the TAIL-PCR, nucleotide sequence isolation and manuscript revision. SA and SMN were responsible for the qPCRs and manuscript revision. MAF designed the qPCR studies, participated in the manuscript revision and data interpretation. JANB has made substantial contributions to experimental design, carried out the TAIL-PCRs and nucleotide sequence isolation and cloning. MCMS was involved in drafting the manuscript and revising it critically for important intellectual content. MFGS has also made substantial contributions to data interpretation, manuscript writing and revision. All authors read and approved the final manuscript.

## Supplementary Material

Additional file 1 **Multiple nucleotide sequence alignment of the E2 family members in cotton**. *G. hirsutum*-1 through 4 correspond to the nucleotide sequences [GenBank:AY082005.1, GenBank:AY082007.1, GenBank:AY082006.1 and GenBank:AY082004.1]. *G. raimondii*, *G. thurberi*, *G. arboreum *and GRDP85 are stored in GenBank under the accession numbers [GenBank:AY082010.1, GenBank:AY082009.1, GenBank:AY082008.1 and GenBank:EU373075.1], respectively. Identical nucleotides are indicated by an asterisk. The alignment was performed using ClustalW with parameters GAP Open of 25, GAP Extension of 0.05 and GAP distances of 2. Start codon, premature stop codon, not spliced intron, qPCR primers, conserved cysteine residue are all indicated by arrows along the multiple sequence alignment. Click here for file

Additional file 2 **Description of cotton genes used in expression pattern analysis by quantitative real-time PCR**. Click here for file

Additional file 3 **Isolation of uceA1.7 from cotton plants by TAIL-PCR**. Fragments were amplified by TAIL-PCR using cotton genomic DNA. (**A**) Amplification products obtained using the UCE2 and W4 primers; (**B**) control reaction with W4 primer; (**C**) control reaction with UCE2 primer. The molecular weights are shown in kb. The arrow indicates the specific amplified fragment of 1,049 bp. Click here for file
