# Supplementary material for: Isolation and functional characterization of a cotton ubiquitination-related promoter and 5'UTR that drives high levels of expression in root and flower tissues
Source: BMC Biotechnol. 2011 Nov 24;11:115. doi: 10.1186/1472-6750-11-115 (PMC3239415; doi:10.1186/1472-6750-11-115)
Supplement: Additional file 1 — Multiple nucleotide sequence alignment of the E2 family members in cotton. G. hirsutum-1 through 4 correspond to the nucleotide sequences [GenBank:AY082005.1, GenBank:AY082007.1, GenBank:AY082006.1 and GenBank:AY082004.1]. G. raimondii, G. thurberi, G. arboreum and GRDP85 are stored in GenBank under the accession numbers [GenBank:AY082010.1, GenBank:AY082009.1, GenBank:AY082008.1 and GenBank:EU373075.1], respectively. Identical nucleotides are indicated by an asterisk. The alignment was performed using ClustalW with parameters GAP Open of 25, GAP Extension of 0.05 and GAP distances of 2. Start codon, premature stop codon, not spliced intron, qPCR primers, conserved cysteine residue are all indicated by arrows along the multiple sequence alignment. [file 1472-6750-11-115-S1.DOC]

G.hirsutum-1 ---------------------GCTCGTTGAGGGCCAAGGAAGGAAG-----------GAA 28

G.hirsutum-4 GGTTGCCGGTGGAATCGACGCGCTCGTTGAGGGCCAAGGAAGGAAG-----------GAG 49

G.arboreum -------------TCAAACTTTCCCGTCTCATATCTAATCCCCAATTTCTCTCTTCTGCT 47

G.hirsutum-3 -------------TCAAACTTTCCCGTCTCATATCTAATCCCCAATTTCTCTCTTCTGCT 47

G.raimondii -------------TCAAACTTTCCCGTCTCATATCTAATCCCCAATTTCTCTCTTTTGCT 47

G.hirsutum-2 -------------TCAAACTTTCCCGTCTCATATCTAATCCCCAATTTCTCTCTTCTGCT 47

G.thurberi -------------TCAAACTTTCCCGTCTCATATCTAATCCCCAATTTCTCTCTTTTTCT 47

GDRP85 ---------GGGGGGAGCTATTCCCAGCGGACTACTAACACAAAAT-------------- 37

* * * * **

G.hirsutum-1 GAAGAGAGAGAGAGAGATAAAAAGGAGAATCTGCTTCAAAGTTTCAAACTTTCCC----- 83

G.hirsutum-4 GAAGAGAGAGAGAGAGATAAAAAGGAGAATCTGCTTCAAAGTTTCAAACTTTCCC----- 104

G.arboreum CTAGGTATATTTTCCTTTTTTTTTCACAACTTTCTTCTTCAAGCTTTACTTTCACCGTGG 107

G.hirsutum-3 CTAGGTATATTTTCCTTTTTTTT-CACAACTTTCTTCTTCAAGCTTTACTTTCACCGTGG 106

G.raimondii CTAGGTATATTTTCTTTTTTCTTTCACAACTTTCTTCTTCAATCTTTACTTTCACCGCGG 107

G.hirsutum-2 CTAGGTATATTTTCTTTTTTCTTTCACAACTTTCTTCTTCAATCTTTACTTTCACTGCAG 107

G.thurberi CTAGGTATATTTTCTTTTTTCTTTCACAGCTTTCTTCTTCAATCTTTACTTTCACTGCGG 107

GDRP85 -----AAAAATTAGCCCAGGACAGGAGGATCTTCTCAGCTACT----------------- 75

* * * * **

G.hirsutum-1 ------------GTCTCATATCTAATCCCCAATTTCTCTCTTCTGCTCTAGGTTCCTTCC 131

G.hirsutum-4 ------------GTCTCATATCTAATCCCCAATTTCTCTCTTCTGCTCTAGGTTCCTTCC 152

G.arboreum TTGCTATATATTGTTCTTTATGTGATGGTTTTTT-CTGATTTTGCCTTCAGGTTCCTTCC 166

G.hirsutum-3 TTGCTATATATTGTTCTTTATGTGATGGTTTTTT-CTGATTTTGCCTTCAGGTTCCTTCC 165

G.raimondii TTGCTATATATTGTTCTTCATGTGATGGTTTTTTTCTGATTTTGTCTTCAGGTTCCTTCC 167

G.hirsutum-2 TTGCTATATATTGTTCTTCATGTGATGGTTTTTT-CTGATTTTGTCTTCAGGTTCCTTCC 166

G.thurberi TTGCTATATATTGTTCTTCATGTGATGGTTTTTT-CTGATTTTGTCTTCAGGTTCCTTCC 166

GDRP85 ---------------------------------------TTTTCTCCTCAAAATTTCTCC 96

** * * * ***

G.hirsutum-1 TGTCCTCTTAGGATTCTTGTGGTCTGTTCAAAGCGTC**ATG**GCCTCGAAGCGGATTTTGAA 191

G.hirsutum-4 TGTTCTCTTAGGATTCTTGTGGTCTGTTCAAAGCGTC**ATG**GCATCGAAGCGGATTTTGAA 212

G.arboreum TGTTCTCTTAGGATTCTTGTGGTCTGTTCAAAGCGTC**ATG**GCATCGAAGCGGATTTTGAA 226

G.hirsutum-3 TGTTCTCTTAGGATTCTTGTGGTCTGTTCAAAGCGTC**ATG**GCATCGAAGCGGATTTTGAA 225

G.raimondii TGTCCTCTTAGGATTCTTGTGGTCTGTTCAAAGCGTC**ATG**GCCTCGAAGCGGATTTTGAA 227

G.hirsutum-2 TGTCCTCTTAGGATTCTTGTGGTCTGTTCAAAGCGTC**ATG**GCCTCGAAGCGGATTTTGAA 226

G.thurberi TGTCCTCTTAGGATACTTGTGGTCTGTTCAAAGCGTC**ATG**GCCTCGAAGCGGATTTTGAA 226

GDRP85 AATTAGCTCTGGTTT-----------------AGGCG**ATG**GCTTCAAAGCGGATCTTGAA 139

* ** ** * * ***** ** ******** *****

↑ **START CODON**

G.hirsutum-1 GGAACTCAAGGATTTGCAAAAGGATCCACCCACTTCTTGCAGTGCAGG------------ 239

G.hirsutum-4 GGAACTCAAGGATTTGCAAAAGGATCCACCCACTTCTTGCAGTGCAGG------------ 260

G.arboreum GGAACTCAAGGATTTGCAAAAGGATCCACCCACTTCTTGCAGTGCAGGTCCCTTTTCTTT 286

G.hirsutum-3 GGAACTCAAGGATTTGCAAAAGGATCCACCCACTTCTTGCAGTGCAGGTCCCTTTTCTTT 285

G.raimondii GGAACTCAAGGATTTGCAAAAGGATCCACCCACTTCTTGCAGTGCAGGTTCCTTTTCTTT 287

G.hirsutum-2 GGAACTCAAGGATTTGCAAAAGGATCCACCCACTTCTTGCAGTGCAGGTCCCTTTTCTTT 286

G.thurberi GGAACTCAAGGATTTGCAAAAGGATCCACCCACTTCTTGCAGTGCAGGTCCCTTTTCTTT 286

GDRP85 GGAGCTCAAGGATCTACAGAAAGATCCTCCTACCTCTTGCAGTGCAGG------------ 187

*** ********* * ** ** ***** ** ** **************

G.hirsutum-1 ------------------------------------------------------------

G.hirsutum-4 ------------------------------------------------------------

G.arboreum TTCTGGACAGACATAAATTTTATGCCCCTCCTGATAATAACTTCATTTATACCACTTTGT 346

G.hirsutum-3 TTCTGGACAGACATAAATTTTATGCCCCTCCTGATAATAACTTCATTTATACCACTTTGT 345

G.raimondii TTCTGGACATACATAAATTTTATGCCCCTCCTGATAATAACTTCATTTATACCACTTTGT 347

G.hirsutum-2 TTCTGGACATACATAAATTTTATGCCCCTCCTGATAATAACTTCATTTATACCACTTTGT 346

G.thurberi TTCTGGACATACATAAATTTTATGCCCCTCCTGATAATAACTTCATTTATACCACTTTGT 346

GDRP85 ------------------------------------------------------------

G.hirsutum-1 ------------------------------------------------------------

G.hirsutum-4 ------------------------------------------------------------

G.arboreum CTATTTTTCTTTTCTCTGGTTTCATGTACAATACGTGTCATTTATATACCCATGAATTTA 406

G.hirsutum-3 CTATTTTTCTTTTCTCTGGTTTCATGTACAATACGTGTCATTTATATACCCATGAATTTA 405

G.raimondii CTATTTTTCTTTTCTCTGGTTTCATGTACAATACGTGTCATTTATATACCCATGAATGTA 407

G.hirsutum-2 CTATTTTTCTTTTCTCTGGTTTCATGTACAATACGTGTCATTTATATACCCATGAATGTA 406

G.thurberi CTATTTTTCTTTTCTCTGGTTTCATGTACAATACGTGTCATTTATATACCCATGAATGTA 406

GDRP85 ------------------------------------------------------------

G.hirsutum-1 ------------------------------------------------------------

G.hirsutum-4 ------------------------------------------------------------

G.arboreum TTGAATTCCATTTAGGATTATATGTATTTGGCTGTATACGTATAACCTTTGCTCAAGCAA 466

G.hirsutum-3 TTGAATTCCATTTAGGATTATATGTATTTGGCTGTATACGTATAACCTTTGCTCAAGCAA 465

G.raimondii TTGAATTCCATTTAGGATTATATGTATTTGGCTGTATACTTATAACCTGTGCTCAAGCAA 467

G.hirsutum-2 TTGAATTCCATTTAGGATTATATGTATTTGGCTGTATACTTATAACCTGTGCTCAAGCAA 466

G.thurberi TTGAATTCCATTTAGGATTATATGTATTTGGCTGTATACTTATAACCTGTGCTCAAGCAA 466

GDRP85 ------------------------------------------------------------

G.hirsutum-1 ------------------------------------------------------------

G.hirsutum-4 ------------------------------------------------------------

G.arboreum GCTTACTTTTGTCCATCCCCAGTGCTTATCGCTGAAATTCCTTAACCATGGATGCTGAGG 526

G.hirsutum-3 GCTTACTTTTGTCCATCCCCAGTGCTTATCGCTGAAATTCCTTAACCATGGATGCTGAGG 525

G.raimondii GCTTGCTTTTGTCCATCCCCAGTGCTTATCGCTGAAATTCCTTAACCATGGATGCTGAGG 527

G.hirsutum-2 GCTTGCTTTTGTCCATCCCCAGTGCTTATCGCTGAAATTCCGTAACCATGGATGCTGAGG 526

G.thurberi GCTTGCTTTTGTCCATCCCCAGTGCTTATCGCTGAAATTCCTTAACCATGGATGCTGAGG 526

GDRP85 ------------------------------------------------------------

G.hirsutum-1 ------------------------------------------------------------

G.hirsutum-4 ------------------------------------------------------------

G.arboreum GTCGGACCCTCAACATGTCGAGGTTTTGTCTCTCTGTCGAGAGCGCTGATGGGTTCACAA 586

G.hirsutum-3 GTCGGACCCTCAACATGTCGAGGTTTTGTCTCTCTGTCGAGAGCGCTGATGGGTTCACAA 585

G.raimondii GTCGGACCCTCAACATGTCGAGGTTTTGTCTCTCTGTCGAGAGCGCTGATGGGTTCACAA 587

G.hirsutum-2 GTCGGACCCTCAACATGTCGAGGTTTTGTCTCTCTGTCGAGAGCGCTGATGGGTTCACAA 586

G.thurberi GTCGGACCCTCAACATGTCGAGGTTTTGTCTCTCTGTCGAGAGCGCTGATGGGTTCACAA 586

GDRP85 ------------------------------------------------------------

G.hirsutum-1 ------------------------------------------------------------

G.hirsutum-4 ------------------------------------------------------------

G.arboreum AGGGGGTGCGCCTATCGGGATGCCTGAATAGGCAGGAGCCATAGTTGCTGAAGAGCGAGC 646

G.hirsutum-3 AGGGGGTGCGCCTATCGGGATGCCTGAATAGGCAGGAGCCATAGTTGCTGAAGAGCGAGC 645

G.raimondii AGCAGGTGCGCGTCTCGGGATGCCTGAATAGGCAGGAGCCAGAGTTACTGAAGAGCGAGC 647

G.hirsutum-2 AGCAGGTGCGCGTCTCGGGATGCCTGAATAGGCAGGAGCCAGAGTTACTGAAGAGCGAGC 646

G.thurberi AGCAGGTGCGCGTCTCGGGATGCCTGAATAGGCAGGAGCCAGAGTTACTGAAGAGCGAGC 646

GDRP85 ------------------------------------------------------------

G.hirsutum-1 ------------------------------------------------------------

G.hirsutum-4 ------------------------------------------------------------

G.arboreum ACATGGTGCCATGGTTGCCGAAAAGCAAGCTAATGGCAAGGCTCGAACTCAAGACCTCTA 706

G.hirsutum-3 ACATGGTGCCATGGTTGCCGAAAAGCAAGCTAATGGCAAGGCTCGAACTCAAGACCTCTA 705

G.raimondii ACATGGTGCCATGGTTGCCGAAAAGCTAGCTAATGGCAAGGCTCGAACTCAAGACCTCTA 707

G.hirsutum-2 ACATGGTGCCATGGTTGCCGAAAAGCTAGCTAATGGCAAGGCTCGAACTCAAGACCTCTA 706

G.thurberi ACATGGTGCCATGGTTGCCGAAAAGCTAGCTAATGGCAAGGCTCGAACTCAAGACCTCTA 706

GDRP85 ------------------------------------------------------------

G.hirsutum-1 ------------------------------------------------------------

G.hirsutum-4 ------------------------------------------------------------

G.arboreum CTATATAGAATTCCTTGAGGGAGCCAGGGTACGGCTTGGGCTAGTGATCCTCAACAAAGT 766

G.hirsutum-3 CTATATAGAATTCCTTGAGGGAGCCAGGGTACGGCTTGGGCTAGTGATCCTCAACAAAGT 765

G.raimondii CTATATAGAAGTCCTTGAGGGAGCCAGGGTACGGCTTGGGCTAGTGACCCTCAACAATGT 767

G.hirsutum-2 CTATATAGAAGTCCTTGAGGGAGCCAGGGTACGGCTTGGGCTAGTGACCCTCAACAATGT 766

G.thurberi CTATATAGAAGTCCTTGAGGGAGCCAGGGTACGGCTTGGGCTAGTGACCCTCAACAAAGT 766

GDRP85 ------------------------------------------------------------

G.hirsutum-1 ------------------------------------------------------------

G.hirsutum-4 ------------------------------------------------------------

G.arboreum AGCATGGGTCCGAAGTCCTTAAGTTTTTAGTCTACAAAATTTTCAAGTCCCATTCTTTAG 826

G.hirsutum-3 AGCATGGGTCCGAAGTCCTTAAGTTTTTAGTCTACAAAATTTTCAAGTCACATTCTTTAG 825

G.raimondii AGCATGGGTCCGAAGTCCCTAAGTTTTTAGTCTACAAAATTTTCGATTCACATTCTTTAG 827

G.hirsutum-2 AGCATGGGTCCGAAGTCCCTAAGTTTTTAGTCTACAAAATTTTCGATTCGCATTCTTTAG 826

G.thurberi AGCATGGGTCCGAAGTCCCTAAGTTTTTAGTCTACAAAATTTTCGATTCACATTCTTTAG 826

GDRP85 ------------------------------------------------------------

G.hirsutum-1 ------------------------------------------------------------

G.hirsutum-4 ------------------------------------------------------------

G.arboreum ACCTAACTTTTGTCATGTGATGCTCTATTATGTCACTAACCTCGGGATGCTTGCACTTTT 886

G.hirsutum-3 ACCTAACTTTTGTCATGTGATGCTCTATTATGTCACTAACCTCGGGATGCTTGCACTTTT 885

G.raimondii ACCTAACTTT-GTCATGTGATGCTCTATTATGTCACTAACCTCGGGATGCTTGCACTTTT 886

G.hirsutum-2 ACCTAACTTT-GTCATGTGATGCTCTATTATGTCACTAACCTCGGGATGCTTGCACTTTT 885

G.thurberi ACCTAACTTT-GTCATGTGATGCTCTATTATGTCACTAACCTCGGGATGCTTGCACTTTT 885

GDRP85 ------------------------------------------------------------

G.hirsutum-1 --------------ACCTGTAGCTGAAGACATGTTTCATTGGCAAGCAACAATCATGGGC 285

G.hirsutum-4 --------------TCCTGTAGCTGAAGACATGTTTCATTGGCAAGCAACACTCATGGGC 306

G.arboreum TTT--TGTGATAGGTCCTGTAGCTGAAGACATGTTTCATTGGCAAGCAACACTCATGGGC 944

G.hirsutum-3 TTT--TGTGATAGGTCCTGTAGCTGAAGACATGTTTCATTGGCAAGCAACACTCATGGGC 943

G.raimondii TTTTTTGTGATAGGACCTGTAGCTGAAGACATGTTTCATTGGCAAGCAACAATCATGGGC 946

G.hirsutum-2 TTTTTTGTGATAGGACCTGTAGCTGAAGACATGTTTCATTGGCAAGCAACAATCATGGGC 945

G.thurberi TTTTTTGTGATAGGACCTGTAGCTGAAGACATGTTTCATTGGCAAGCAACAATCATGGGC 945

GDRP85 --------------CCCTGTTGCTGAGGACATGTTTCATTGGCAAGCGACTATTATGGGT 233

***** ***** ******************** ** * *****

G.hirsutum-1 CCTTCCGATAGCCCTTATGCGGGAGGTGTATTTTTAGTTAGTATTCATTTTCCTCCAGAT 345

G.hirsutum-4 CCTTCCGATAGCCCTTATGCGGGAGGTGTATTTTTAGTTAGTATTCATTTTCCTCCAGAT 366

G.arboreum CCTTCCGATAGCCCTTATGCGGGAGGTGTATTTTTAGTTAGTATTCATTTTCCTCCAGAT 1004

G.hirsutum-3 CCTTCCGATAGCCCTTATGCGGGAGGTGTATTTTTAGTTAGTATTCATTTTCCTCCAGAT 1003

G.raimondii CCTTCCGATAGCCCTTATGCGGGAGGTGTATTTTTAGTTAGTATTCATTTTCCTCCAGAT 1006

G.hirsutum-2 CCTTCCGATAGCCCTTATGCGGGAGGTGTATTTTTAGTTAGTATTCATTTTCCTCCAGAT 1005

G.thurberi CCTTCCGATAGCCCTTATGCGGGAGGTGTATTTTTAGTTAGTATTCATTTTCCTCCAGAT 1005

GDRP85 CCACCTGACAGTCC***ATATGCCGGTGGAGTGTTTC***TAGTCACCATTCATTTCCCTCCGGAC 293

** * ** ** ** ***** ** ** ** *** **** * ******** ***** **

↑ ***qPCR PRIMER SENSE***

G.hirsutum-1 TATCCTTTCAAGCCCCCTAAGG-------------------------------------- 367

G.hirsutum-4 TATCCTTTCAAGCCCCCTAAGG-------------------------------------- 388

G.arboreum TATCCTTTCAAGCCCCCTAAGG-------------------------------------- 1026

G.hirsutum-3 TATCCTTTCAAGCCCCCTAAGG-------------------------------------- 1025

G.raimondii TATCCTTTCAAGCCCCCTAAGG-------------------------------------- 1028

G.hirsutum-2 TATCCTTTCAAGCCCCCTAAGG-------------------------------------- 1027

G.thurberi TATCCTTTCAAGCCCCCTAAGG-------------------------------------- 1027

GDRP85 TATCCATTTAAGCCACCCAAGGGTAAATGGCTTGTGCAAAC***ATTGCTTGGCATTGACATG*** 353

***** ** ***** ** ****

↑ ***qPCR PRIMER ANTISENSE***

↑ **INTRON FOR ALL OTHER E2 HOMOLOGS**

G.hirsutum-1 ---------------T-------------------------------------------- 368

G.hirsutum-4 ---------------T-------------------------------------------- 389

G.arboreum ---------------TAGTTTGCTTCACCTGGATCGTTTTATTATCCCTTTTCTGTGTGC 1071

G.hirsutum-3 ---------------TAGTTTGCTTCACCTGGATCGTTTTATTATCCCTTTTCTGTGTGC 1070

G.raimondii ---------------TAGTTTGCTTCACCTGGATCGTTTTATTATCCCTTTTCTGTGTGT 1073

G.hirsutum-2 ---------------TAGTTTGCTTCACCTGGATCGTTTTATTATCCCTTTTCTCTGTGT 1072

G.thurberi ---------------TAGTTTGCTTCACCTGGATCGTTTTATTATCCCTTTTCTGTGTGC 1072

GDRP85 ***A***GTAAAAGC***TAA***TTCTTTGTAGCTTTTGTTAGCTTATTCTGTTACACAATTATTTTTCTT 413

*

↑ ***PREMATURE STOP CODON***

G.hirsutum-1 ------------------------------------------------------------

G.hirsutum-4 ------------------------------------------------------------

G.arboreum GTAGAATGT-------------------------TGTTTGAGTGGTACGTACTCTTTTCA 1106

G.hirsutum-3 GTAGAATGT-------------------------TGTTTGAGTGGTACGTACTCTTTTCA 1105

G.raimondii GTGAAATGT-------------------------TGTTTGAGTGGTACGTCCTCTTTTCA 1108

G.hirsutum-2 GTGAAATGT-------------------------TGTTTGAGTGGTACGTCCTCTTTTCA 1107

G.thurberi GTGAAATGT-------------------------TGTTTGAGTGGTACGTCCTCTTTTCA 1107

GDRP85 TTGGCATGTATGCATGCACCACTGTGCATGCATATGCATGCTAGGTGTCTAATGGTTTAG 473

G.hirsutum-1 ------------------------------------------------------------

G.hirsutum-4 ------------------------------------------------------------

G.arboreum TGGATAA----------------------------------------------------- 1113

G.hirsutum-3 TGGATAA----------------------------------------------------- 1112

G.raimondii TGGATAA----------------------------------------------------- 1115

G.hirsutum-2 TGGATAA----------------------------------------------------- 1114

G.thurberi TGGATAA----------------------------------------------------- 1114

GDRP85 TTTATATGTGAAAGGCTATTATTAGGCGGTTTTCAGTGCCAAAGTTTACATGATCTATTG 533

G.hirsutum-1 -------------------------------TGCATTTAGGACCAAGGTTTTCCATCCAA 397

G.hirsutum-4 -------------------------------TGCATTTAGGACCAAGGTTTTCCATCCAA 418

G.arboreum -----------TGCTGCCATCATGTTTAGGTTGCATTTAGGACCAAGGTTTTCCATCCAA 1162

G.hirsutum-3 -----------TGCTGCCATCATGTTTAGGTTGCATTTAGGACCAAGGTTTTCCATCCAA 1161

G.raimondii -----------TGCTGCCATCATGTTTAGGTTGCATTTAGGACCAAGGTTTTCCATCCAA 1164

G.hirsutum-2 -----------TGCTGCCATCATGTTTAGGTTGCATTTAGGACCAAGGTTTTCCATCCAA 1163

G.thurberi -----------TGCTGCCATCATGTTTAGGTTGCATTTAGGACCAAGGTTTTCCATCCAA 1163

GDRP85 TTATTTTTAACTTTTGTAAACAGTCTCAGGTTGCATTCAGGACAAAGGTCTTTCACCCTA 593

****** ***** ***** ** ** ** *

G.hirsutum-1 ACATCAATAGCAATGGGAGCATT***TGT***CTTGATATCCTAAAAGAACAGTGGAGTCCAGCCC 457

G.hirsutum-4 ACATCAATAGCAATGGGAGCATT***TGT***CTTGATATCCTAAAAGAACAGTGGAGTCCAGCCC 478

G.arboreum ACATCAATAGCAATGGGAGCATT***TGT***CTTGATATCCTAAAAGAACAGTGGAGTCCAGCCC 1222

G.hirsutum-3 ACATCAATAGCAATGGGAGCATT***TGT***CTTGATATCCTAAAAGAACAGTGGAGTCCAGCCC 1221

G.raimondii ACATCAATAGCAATGGGAGCATT***TGT***CTTGATATCCTAAAAGAACAGTGGAGTCCAGCCC 1224

G.hirsutum-2 ACATCAATAGCAATGGGAGCATT***TGT***CTTGATATCCTAAAAGAACAGTGGAGTCCAGCCC 1223

G.thurberi ACATCAATAGCAATGGGAGCATT***TGT***CTTGATATCCTAAAAGAACAGTGGAGTCCAGCCC 1223

GDRP85 ATATTAACAGCAATGGCAGCATT***TGC***CTCGATATTTTGAAGGAGCAGTGGAGCCCTGCCC 653

* ** ** ******** ******** ** ***** * ** ** ******** ** ****

↑ ***CONSERVED ACTIVE SITE CYSTEINE CODON***

G.hirsutum-1 TAACCATTTCCAAGGTT------------------------------------------- 474

G.hirsutum-4 TAACCATTTCCAAGGTT------------------------------------------- 495

G.arboreum TAACCATTTCCAAGGTTTGACCTTACTCCTGCTAACCTTATGGTATTTCATACACATGCA 1282

G.hirsutum-3 TAACCATTTCCAAGGTTTGACCTTACTCCTGCTAACCTTATGGTATTTCATACACATGCA 1281

G.raimondii TAACCATTTCCAAGGTTTGACCTTACTCCTGCTAACCTTATGGTATTTCATACACATGCA 1284

G.hirsutum-2 TAACCATTTCCAAGGTTTGACCTTACTCCTGTTAACCTTATGGTATTTCATACACATGCA 1283

G.thurberi TAACCATTTCCAAGGTTTGACCTTACTCCTGCTAACCTTATGGTATTTCATACACATGCA 1283

GDRP85 TCACCATATCCAAGGTA------------------------------------------- 670

* ***** ********

G.hirsutum-1 ------------------------------------------------------------

G.hirsutum-4 ------------------------------------------------------------

G.arboreum GAATCTCGGTTTTTGGCTGACTATGGCTGCACAAAAGTTTCTCTGGTGTCACTTTTAATT 1342

G.hirsutum-3 GAATCTCGGTTTTTGGCTGACTATGGCTGCACAAAAGTTTCTCTGGTGTCACTTTTAATT 1341

G.raimondii GAATCTCGGTTTTTGGCCGACTATGGCTGCACAAAAGTTTCTCTGGTGTTACTTTTAAAT 1344

G.hirsutum-2 GAATCTTGGTTTTTGGCCGACTATGGCTGCACAAAAGTTTCTCTGGTGTTACTTTTAAAT 1343

G.thurberi GAATCTCGGTTTTTGGCTGACTATGGCTGCACAAAAGTTTCTCTGGTATTACTTTTAAAT 1343

GDRP85 ------------------------------------------------------------

G.hirsutum-1 ------------------------------------------------------------

G.hirsutum-4 ------------------------------------------------------------

G.arboreum GATTTCAATATCCTTGGGTAAAATTACTTTCTATTGGTTATAAGTTAGACTTGCATTTTA 1402

G.hirsutum-3 GATTTCAATATCCTTGGGTAAAATTACTTTCTATTGGTTATAAGTTAGACTTGCATTTTA 1401

G.raimondii GATTTCAATATCCTTGGGTAAAATTAGTTTCTATTGGTTATAAGTTAGACTTGCATTTTA 1404

G.hirsutum-2 GATTTCAATATCCTTGGGTAAAATTACTTTCTATTGGTTATAAGTTAGACTTGCATTTTA 1403

G.thurberi GATTTCAATATCCTTGGGTAAAATTACTTTCTATTGGTTATAAGTTAGACTTGCATTTTA 1403

GDRP85 ------------------------------------------------------------

G.hirsutum-1 ------------------------------------------------------------

G.hirsutum-4 ------------------------------------------------------------

G.arboreum CGAGTTTGTACGCGTTTCCAACATACGAACAATCACTGCCGTTACTCCCCGAGTTTAATT 1462

G.hirsutum-3 CGAGTTTGTACGCGTTTCCAACATACGAACAATCACTGCCGTTACTCCCCGAGTTTAATT 1461

G.raimondii CGAGTTTGTATGCATTTCCAACACACGAACAATCACTACCGTTACTCCCCGAGTTTAATT 1464

G.hirsutum-2 CGAGTTTGTATGCGTTTCCAACACACGAACAATCACTACAGTTACTCCCCGAGTTTAATT 1463

G.thurberi CGAGTTTGTATGCGTTTCCAACACTCGAACAATCACTACCGTTACTCCCCGAGTTTAATT 1463

GDRP85 ------------------------------------------------------------

G.hirsutum-1 ------------------------------------------------------------

G.hirsutum-4 ------------------------------------------------------------

G.arboreum TTCTTTCTGGGAATTGAATAATTAATATCAGAAGAGAATTTCTTTCTGGGAATTGAATAA 1522

G.hirsutum-3 TTCTTTCTGGGAATTGAATAATTAATATCAGAAGAGAATTTCTTTCTGGGAATTGAATAA 1521

G.raimondii --CTTTCTGGGAATTGAATAATTAATATCAGAAGAGAAAGACCAACC-----TTGATCAA 1517

G.hirsutum-2 --CTTTCTGGGAATTGAATAATTAATATCAGAAGAGAAAGACCAACC-----TTGATTAA 1516

G.thurberi --CTTTCTGGGAATTGAATAATTAATATCAGAAGAGAAAGACCAACC-----TTGATCAA 1516

GDRP85 ------------------------------------------------------------

G.hirsutum-1 ------------------------------------------------------------

G.hirsutum-4 ------------------------------------------------------------

G.arboreum TTAATATCAGAAGAGAAAGACCAACCTTGATCA----AATATCATTGGCATTATATTGTC 1578

G.hirsutum-3 TTAATATCAGAAGAGAAAGACCAACCTTGATCA----AATATCATTGGCATTATATTGTC 1577

G.raimondii ATATCATTGGCA-ATACGCACCAACTTTAGCCACTTTAGAAAATGAACCATTATATTGTT 1576

G.hirsutum-2 ATATCATTGGCA-ATACGCACCAACTTTAGCCACTTTAGAAGATGAACTATTATATTGTT 1575

G.thurberi ATATCATTGGCA-ATAAGCACCAACTTTAGCCACTTTAGAAGATGAACCATTATATTGTT 1575

GDRP85 ------------------------------------------------------------

G.hirsutum-1 ------------------------------------------------------------

G.hirsutum-4 ------------------------------------------------------------

G.arboreum ATTGTTACTATCCTTCTTTGCATTGGCTTCTGCTGCTGCTTGGAAATTTAAGTAATTTTT 1638

G.hirsutum-3 ATTGTTACTATCCTTCTTTGCATTGGCTTCTGCTGCTGCTTGGAAATTTAAGTAATTTTT 1637

G.raimondii GTTGTTACTATCCTTCTTTGCATTGGCTTCTGCCGCTGATTGGAAATTTAAGTAATTTAT 1636

G.hirsutum-2 GTTGTTACTATCCTTCTTTGCATTGGCTTCTGCCGCTGATTGGAAATAAAAGAGTTTAA- 1634

G.thurberi GTTGTTACTATCCTTCTTTGCATTGGCTTCTGCCGCTGATTGGAAATTTAAGTAATTTAT 1635

GDRP85 ------------------------------------------------------------

G.hirsutum-1 ------------------------------------------------------------

G.hirsutum-4 ------------------------------------------------------------

G.arboreum GGTCTTAAATATCATTAGAGCTTGTCTTAGAATAAGATAACATGGTAAAAAAAAAAAAAA 1698

G.hirsutum-3 GGTCTTAAATATCATTAGAGCTTGTCTTAGAATAAGATAACATGGTAAAAAAAAAAAAA- 1696

G.raimondii GGTCTTAAATATCATTAGAGCTTGTCTTAGAATAAGATAACATGGTAAAAAAAGAGTT-- 1694

G.hirsutum-2 ------------------------------------------------------------

G.thurberi GGTCTTAAATATCATTAGAGCTTGTCTTAGAATAAGATAACATGGTAAAAAAAGAGTTT- 1694

GDRP85 ------------------------------------------------------------

G.hirsutum-1 ---------------------------CTGCTCTCGATCTGCTCGTTGTTGACTGATCCA 507

G.hirsutum-4 ---------------------------CTGCTCTCGATCTGCTCGTTGTTGACTGATCCA 528

G.arboreum AGTTTAACTCCAATGCAAAACCAGGTTCTGCTCTCGATCTGCTCGTTGTTGACTGATCCA 1758

G.hirsutum-3 -GTTTAACTCCAATGCAAAACCAGGTTCTGCTCTCGATCTGCTCGTTGTTGACTGATCCA 1755

G.raimondii -------CTCCAATGCAAAACCAGGTTCTGCTCTCGATCTGCTCGTTGTTGACTGATCCA 1747

G.hirsutum-2 -------CTCCAATGCGAAACCAGGTTCTGCTCTCGATCTGCTCGTTGTTGACTGATCCA 1687

G.thurberi ----TAACTCCAATGCGAAACCAGGTTCTGCTCTCGATCTGCTCGTTGTTGACTGATCCA 1750

GDRP85 ---------------------------TTGCTCTCAATCTGCTCACTTTTGACGGACCCA 703

******* ******** * ***** ** ***

G.hirsutum-1 AACCCTGATGACCCACTTGTTCTGGAGATTGCGCACATGTATAAGACTGATCGGGCAAAG 567

G.hirsutum-4 AACCCTGATGACCCACTTGTTCCGGAGATTGCACACATGTATAAGACTGATCGGGCAAAG 588

G.arboreum AACCCTGATGACCCACTTGTTCCGGAGATTGCACACATGTATAAGACTGATCGGGCAAAG 1818

G.hirsutum-3 AACCCTGATGACCCACTTGTTCCGGAGATTGCACACATGTATAAGACTGATCGGGCAAAG 1815

G.raimondii AACCCTGATGACCCACTTGTTCCGGAGATTGCGCACATGTATAAGACTGATCGGGCAAAG 1807

G.hirsutum-2 AACCCTGATGACCCACTTGTTCTGGAGATTGCGCACATGTATAAGACTGATCGGGCAAAG 1747

G.thurberi AACCCTGATGACCCACTTGTTCCGGAGATTGCGCACATGTATAAGACTGATCGGGCAAAG 1810

GDRP85 AATCCCGATGATCCCTTGGTGCCAGAGATTGCCCACATGTACAAGACCGACAGGGCTAAG 763

** ** ***** ** * ** * ******** ******** ***** ** **** ***

G.hirsutum-1 TATGAAGCTACAGCACGTGGCTGGACCCAGAAGTATGCCATGGGATGATGGTGGTTAGTG 627

G.hirsutum-4 TACGAAGCGACAGCATGTGGCTGGACCCAGAAGTATGCCATGGGATGATGGTGGTTAGTG 648

G.arboreum TACGAAGCGACAGCACGTGGCTGGACCCAGAAGTATGCCATGGGATGATGGTGGTTAGTG 1878

G.hirsutum-3 TACGAAGCGACAGCATGTGGCTGGACCCAGAAGTATGCCATGGGATGATGGTGGTTAGTG 1875

G.raimondii TATGAAGCTACAGCACGTGGCTGGACCCAGAAGTATGCTATGGGATGATGGTGGTTAGTG 1867

G.hirsutum-2 TATGAAGCTACAGCACGTGGCTGGACCCAGAAGTATGCCATGGGATGATGGTGGTTAGTG 1807

G.thurberi TATGAAGCTACAGCACGTGGCTGGACCCAGAAGTATGCCATGGGATGATGGTGGTTAGTG 1870

GDRP85 TACGAGACAACTGCTCGGAGCTGGACCCAGAAGTATGCTATGGGTTAGTGTGCTAATATG 823

** ** * ** ** * ******************* ***** * ** **

G.hirsutum-1 GGTAATA-CGTATTGGCTTTTGTACGATGCCATGTATGTGTTAAAACCTTCATATATTTT 686

G.hirsutum-4 TGTAATA-CGTATTGGCTTTGGTACGATGCCATATATGTGTTAAAACCTTCATATATTTT 707

G.arboreum TGTAATA-CGTATTGGCTTTGGTACGATGCCATATATGTGTTAAAACCTTCATATATTTT 1937

G.hirsutum-3 TGTAATA-CGTATTGGCTTTGGTACGATGCCATATATGTGTTAAAACCTTCATATATTTT 1934

G.raimondii GGTAATA-CGTATTGGCTTTTGTACGATGCCATGTATGTGTTAAAACCTTCATATGTTTT 1926

G.hirsutum-2 GGTAATA-CGTATTGGCTTTTGTACGATGCCATGTATGTGTTAAAACCTTCATATATTTT 1866

G.thurberi GGTAATA-CGTATTGGCTTTTGTACGATGCCATGTATGTGTTAAAACCTTAATATATTTT 1929

GDRP85 CATAATAATAGGAGGGCTTTATTCCTATGTGGCTTTGATCCTTCAAT-TTAATATGTATG 882

***** ****** * * *** * * * ** ** **** * *

G.hirsutum-1 GGTTTGGGAAGGAACTTGTGATTGTGCTTAGATTTGGATTTGAATTATGTGGACATCTTA 746

G.hirsutum-4 G------------------------------ATTTGGATTTGAATTACGTGGACATCTTA 737

G.arboreum G------------------------------ATTTGGATTTGAATTATGTGGACATCTTA 1967

G.hirsutum-3 G------------------------------ATTTGGATTTGAATTACGTGGACATCTTA 1964

G.raimondii GGTTTGGGAAGGAACTTGTGATTGTGCTTAGATTTGGATTTAAATTATGTGGACATCTTA 1986

G.hirsutum-2 GGTTTGGGAAGGAACTTGTGATTGTGCTTAGATTTGGATTTGAATTATGTGGACATCTTA 1926

G.thurberi GGTTTGGGAAGGAACTTGTGATTGTGCTTAGATTTGGATTTGAATTATGTGGACATCTTA 1989

GDRP85 AATCAATGAAACAGTATG-------------GTGTGATTCTTGTCTCTTCCAACTTATTG 929

* ** * * * ** * **

G.hirsutum-1 TATCAAAGGTGGGTCTATGGTTGTAAGAGAGAATGCCATTCCATTCCATGTCTTTTTATA 806

G.hirsutum-4 TATGAAAGG--------------------------------------------------- 746

G.arboreum TATGAAAGGCATTTCCTA------------------------------------------ 1985

G.hirsutum-3 TATGAAAGGCATTTCCTA------------------------------------------ 1982

G.raimondii TATCAAAGGTGGGTCTATGGTTGTAAG--------------------------------- 2013

G.hirsutum-2 TATCAAAGGTGGGTCTATGGTTGTAAG--------------------------------- 1953

G.thurberi TATCAAAGGTGGGTCTATGGTTGTAAG--------------------------------- 2016

GDRP85 TTACCTGGACCAATCCCCCTCCCCTTAAAAACACCCATTTGTGTTGTCCCCTTAATTGTT 989

* *

G.hirsutum-1 GCTTGTTATATGATGAAGCATTTCCTAAAAAAAAAAAAAAAAAA- 850

G.hirsutum-4 ------------------CATTTCCTAAAAAAAAAAAAAAAAAAA 773

G.arboreum ---------------------------------------------

G.hirsutum-3 ---------------------------------------------

G.raimondii ---------------------------------------------

G.hirsutum-2 ---------------------------------------------

G.thurberi ---------------------------------------------

GDRP85 TGTATGGAAATTATGTTTCAAACTTTGAAAATCCCCCCTTT---- 1030
