# Supplementary material for: Isolation and functional characterization of a cotton ubiquitination-related promoter and 5'UTR that drives high levels of expression in root and flower tissues
Source: BMC Biotechnol. 2011 Nov 24;11:115. doi: 10.1186/1472-6750-11-115 (PMC3239415; doi:10.1186/1472-6750-11-115)
Supplement: Additional file 2 — Description of cotton genes used in expression pattern analysis by quantitative real-time PCR. [file 1472-6750-11-115-S2.DOC]

**Table S1. Description of cotton genes used in expression pattern analysis by quantitative real time PCR**

|  |  |  |  |  |  |  |  |
| --- | --- | --- | --- | --- | --- | --- | --- |
| ***Gene abbreviation*** | ***Accession number*** | ***Orthologue locus*** | ***A. thaliana annotation*** | ***Similarity (e-value)*** | ***Identity (%)*** | ***Gene Size***** | ***Blast alignment*** |
|  |  |  |  |  |  |  |  |
| GhGDRP-85 | EU373075 | − | − | − | − | − | − |
|  |  |  |  |  |  |  |  |
| GhACT4 | AY305726 | At5g09810 | Actin gene family | 6,90E-194 | 86% | 1700 | 1013 |
|  |  |  |  |  |  |  |  |
| *GhFBX6 | DR463903 | At5g15710 | F-box family protein | 2,30E-93 | 79% | 1884 | 567 |
|  |  |  |  |  |  |  |  |
| *GhPP2A1 | DT545658 | At1g59830 | Catalytic subunit of protein phosphatase 2A | 3,30E-110 | 77% | 1301 | 675 |
|  |  |  |  |  |  |  |  |
| *GhUBQ14 | DW505546 | At4g02890 | Polyubiquitin | 0.0 | 80% | 1502 | 510 |
|  |  |  |  |  |  |  |  |

***All cotton sequences were named according the most similar orthologue locus.**

****Size in base pair (bp) of the coding sequence of the orthologue locus in *A. thaliana*.**
